# Unraveling multifaceted contributions of small regulatory RNAs to photomorphogenic development in *Arabidopsis*

**DOI:** 10.1186/s12864-017-3937-6

**Published:** 2017-07-24

**Authors:** Meng-Chun Lin, Huang-Lung Tsai, Sim-Lin Lim, Shih-Tong Jeng, Shu-Hsing Wu

**Affiliations:** 1 Institute of Plant and Microbial Biology, Academia Sinica, Taipei, 11529 Taiwan; 2 Institute of Plant Biology, College of Life Science, National Taiwan University, Taipei, 106 Taiwan

**Keywords:** Light, Small regulatory RNA, Post-transcriptional regulation, Photomorphogenesis

## Abstract

**Background:**

Post-transcriptional control of gene expression mediated by small regulatory RNAs (sRNAs) is vital for growth and development of diverse organisms. The biogenesis of sRNAs is regulated by both positive and negative regulators known to regulate photomorphogenic development. Two microRNAs (miRNAs), miR157 and miR319, also regulate photomorphogenesis. However, genome-wide profiling of sRNAs and their regulation of target genes during photomorphogenesis has been missing. We provide a comprehensive view of sRNA-controlled gene expression in this developmental process.

**Results:**

By profiling sRNAs and the 5′ ends of degraded mRNAs during the first 24 h of photomorphogenic development in *Arabidopsis*, we identified 335 sRNA-mediated mRNA cleavage events in de-etiolating seedlings. These cleavage events are primarily resulted from actions of highly expressed miRNAs and irrelevant to the abundance of target mRNAs. In the light, the expression of the slicer protein gene *ARGONAUTE1* in the miRNA functioning pathway could be fine-tuned by miRNA168a/b. We also found that miR396a/b positively regulates de-etiolation by suppressing *GROWTH REGULATING FACTOR*s. Our results suggest that the miRNAs are required to tune down the target mRNAs and regulate photomorphogenesis.

**Conclusion:**

sRNAs may have a broad impact on gene expression regulation for optimized photomorphogenic development. With both positive and negative regulators under the control of sRNAs, young *Arabidopsis* seedlings can have a timely but not exaggerated developmental adaptation to light.

**Electronic supplementary material:**

The online version of this article (doi:10.1186/s12864-017-3937-6) contains supplementary material, which is available to authorized users.

## Background

Plants have evolved a plethora of morphological alterations to adapt to their surroundings. Photomorphogenesis, or de-etiolation, is one such process when seedlings first experience light irradiation. The rate of hypocotyl elongation decreases in seedlings under light exposure, which allows for the formation of firm structural support for seedlings emerging from the soil surface. Also, the cotyledons open and expand to maximize the area of light perception and to expose the shoot apical meristem for the development of true leaves. Light also triggers the development of chloroplasts for photosynthesis so that plants can utilize light energy for autotrophic growth and development [1–3].

In *Arabidopsis*, photomorphogenesis is under the control of at least three types of photoreceptors, including the red (R)–far-red (FR) light photoreceptor phytochromes (phys), blue light (B) photoreceptor cryptochromes (crys) and the UV-B photoreceptor, UVR8 [3–8]. The perceived light signals trigger signaling cascades that reprogram gene expression for photomorphogenic development. Transcriptional profiling for *Arabidopsis* seedlings exposed to B, FR, R light and the light–dark transition have revealed differential expression of approximately one-third of the genome [9]. The light-regulated genome-wide transcriptomic adjustment requires the actions of transcription factors. One of the most well-characterized transcription factors conveying light signals to changes of gene expression is ELONGATED-HYPOCOTYL 5 (HY5). HY5 is a light-regulated bZIP transcription factor that upregulates the expression of many light-responsive genes during de-etiolation [10]. In addition to activating transcription, light also enhances the translational efficiency of thousands of genes, especially those committed to the translation apparatus and chloroplast functions [11, 12].

Plant small regulatory RNAs (sRNAs) are 20 to 24 nt long and can be classified into microRNAs (miRNAs) and small interfering RNAs (siRNAs) primarily according to different modes of biogenesis. MiRNAs originate from stem-loop structures of primary transcripts, and siRNAs are mostly derived from double-stranded RNAs [13]. Phased siRNAs (phasiRNAs) are a special group of siRNAs generated from mRNAs cleaved by 22-nt miRNAs or siRNAs [14–16]. Plant miRNAs can mediate the cleavage or translation inhibition of target mRNAs, whereas siRNAs function via RNA-dependent DNA methylation (RdDM) for transcriptional gene silencing or post-transcriptional target mRNA cleavage [17–22].

Previous studies have implied that sRNAs are involved in gene expression regulation during de-etiolation. Mutants defective in genes for miRNA biogenesis and functions have altered light responses. For example, in *Arabidopsis*, light hypersensitive phenotypes have been observed to carry mutations in the miRNA processor HYPONASTIC LEAVES 1 (HYL1), the sRNA methyltransferase HUA ENHANCER1 (HEN1), the sRNA transporter HASTY (HST), and the slicer protein ARGONAUTE1 (AGO1) [23–25]. A negative regulator of photomorphogenesis, CONSTITUTIVE PHOTOMORPHOGENESIS 1 (COP1), can protect HYL1 against degradation, thereby leading to a stabilized miRNA pool [26]. Transcripts of the positive regulator HY5 and negative regulator TEOSINTE BRANCHED 1, CYCLOIDEA AND PCF TRANSCRIPTION FACTORs (TCPs) of photomorphogenesis were shown to be under regulation by miR157d and miR319, respectively [25]. In addition, HY5 was found to bind to promoter regions of at least 8 miRNAs (*MIR*) loci and required for the accumulation of miR156d, miR402, miR408, miR775 and miR858 [27]. These studies provide a glimpse into photomorphogenic development shaped by the actions of a few sRNAs. A global investigation of sRNAs and their targets would greatly help in assessing the impact of post-transcriptional regulation in photomorphogenic development. However, such information is currently missing in de-etiolating seedlings.

In this study, we profiled sRNAs at 6 times during the first 24 h of *Arabidopsis* photomorphogenic development. We also sequenced 5′ ends of degraded mRNAs (degradome) in both dark- and light-grown seedlings to reveal sRNA-mediated cleavage of mRNAs during de-etiolation. Pairwise studies of sRNAs and their target mRNAs indicated that a high sRNA-to-target ratio is a key determinant for successful mRNA target repression by sRNAs. The high ratio is mainly contributed by the abundance of sRNAs. A total of 335 sRNA-target mRNA regulatory pairs were identified in de-etiolating seedlings, with several sRNAs demonstrated to regulate photomorphogenesis. The action of miR168 leads to reduced expression of *AGO1* under light, thereby offering a feedback regulation of miRNA functioning during de-etiolation. miR396 were identified to act as positive regulators of photomorphogenesis. In addition, we revealed that some 24-nt siRNAs had potential to cause target cleavage in de-etiolating seedlings. Our data indicate that sRNAs function in multiple regulatory circuits for optimized seedling growth under light illumination.

## Results

### The expression and actions of small RNAs in de-etiolating *Arabidopsis* seedlings

**Fig. 1:**
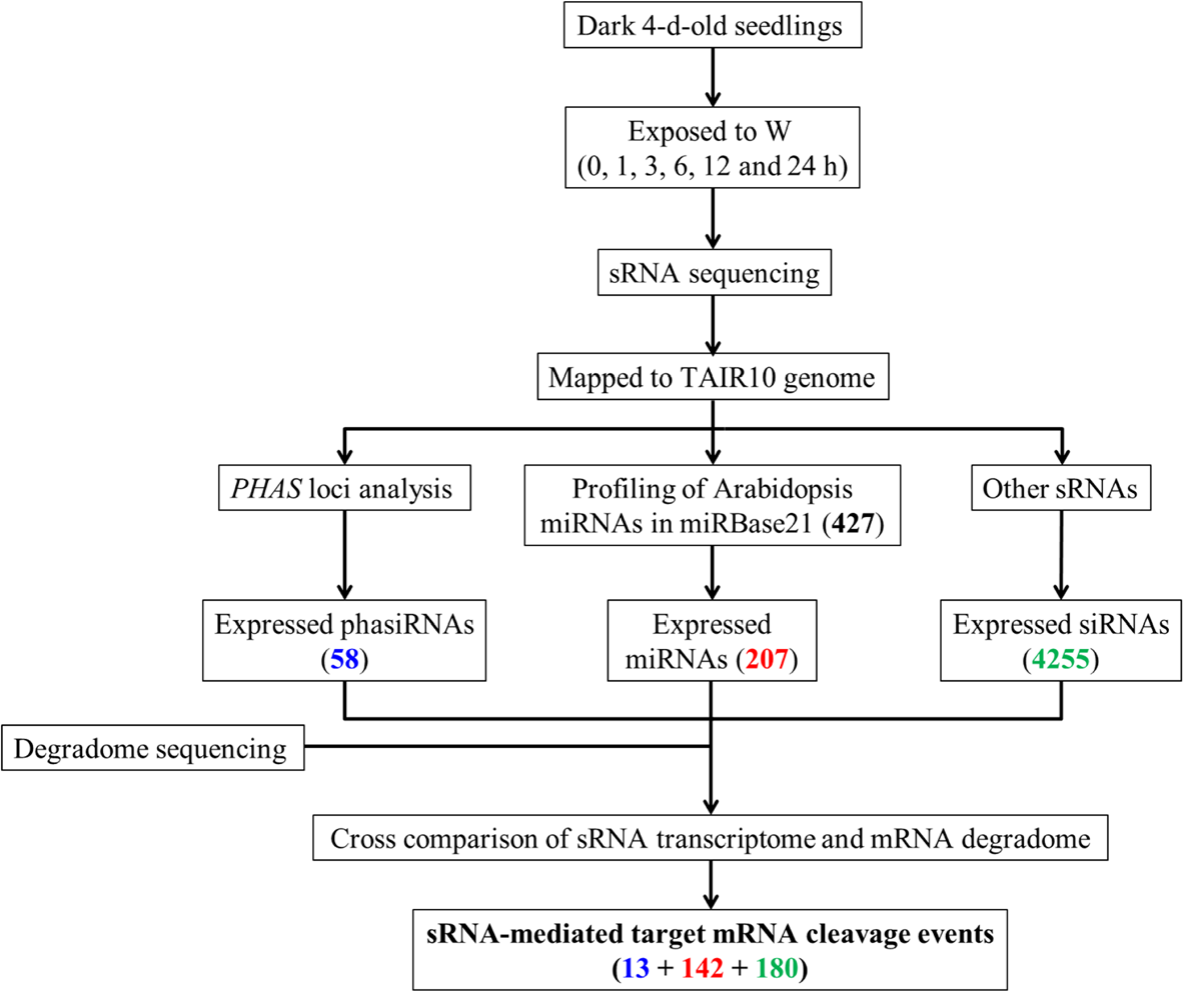
Pipeline for investigating sRNA-mediated regulation in de-etiolating *Arabidopsis* seedlings. Four-d-old dark-grown seedlings (W0 h) were exposed to continuous white light (W; 100 μE) for 1, 3, 6, 12 and 24 h; 18–22 million (M) reads per library were acquired. Degradome sequencing for dark- (Dark) and light- (equally pooled from the five light-treated time points) grown seedlings were used for identifying sRNA-mediated cleavage of target mRNA. Colored numbers in parentheses indicate the number of sRNAs/targets passing the corresponding filtering criteria

We first used deep sequencing to survey the sRNAs in de-etiolating *Arabidopsis* seedlings. The sRNAs were isolated from 4-d-old dark-grown (W0 h) seedlings and seedlings that were further treated with continuous white light irradiation for 1 to 24 h (W1 h to W24 h) and subjected to deep sequencing (Fig. 1). Approximately 18–22 million reads were obtained for each sample in 3 biological replicates. For each dataset, 94–98% of the filtered reads (see methods) could be mapped to the TAIR10 genome (Additional file 1: Table S1). We first analyzed miRNAs and phasiRNAs, as they are frequently studied groups of plant sRNAs. Among the 427 *Arabidopsis* miRNAs annotated in miRBase 21 [28], 207 (48.5%) are considered expressed (see methods for criteria) (Additional file 1: Table S2). Overall 58 phasiRNAs derived from 12 phasiRNA-generating loci (*PHAS*, or trans-acting siRNA-generating loci, *TAS*) [14] were expressed in de-etiolating seedlings (Table 1 and Additional file 1: Table S3). In addition to miRNAs and phasiRNAs, 4255 20–24 nt siRNAs were defined expressed in this developmental stage (Additional file 1: Table S4).

**Table 1:** Expressed *PHAS* loci in de-etiolating seedlings

*PHAS* locus	Gene	Chromosome	phasiR trigger	Start	End	n	k	*p*-value
*At1g62910*	*RFL9 (PPR)*	1	TAS2 3′-D6 (−)	23,299,624	23,299,875	77	14	2.0 × 10^−7^
*At1g63130*	*RPF6*	1	ta-siR2140	23,413,391	23,413,642	13	7	7.3 × 10^−7^
*At1g50055*	*TAS1B*	1	miR173	18,549,441	18,549,692	60	12	9.7 × 10^−7^
*At1g63070*	*PPR*	1	ta-siR2140	23,386,420	23,386,671	50	11	1.3 × 10^−6^
*At1g63150*	*	1	TAS2 3′-D6 (−)	23,420,003	23,420,254	80	12	2.7 × 10^−5^
*At1g62930*	*RPF3 (PPR)*	1	miR161.1	23,307,125	23,307,466	99	13	4.5 × 10^−5^
*At1g63080*	*PPR*	1	ta-siR2140	23,389,990	23,390,241	58	10	5.5 × 10^−5^
*At1g62590*	*PPR-AC*	1	TAS2 3′-D6 (−)	23,178,438	23,178,689	27	7	7.1 × 10^−5^
*At2g39681*	*TAS2*	2	miR173	16,539,919	16,540,170	12	9	1.9 × 10^−9^
*At2g39675*	*TAS1C*	2	miR173	16,537,860	16,538,111	10	7	1.5 × 10^−8^
*At2g27400*	*TAS1A*	2	miR173	11,722,009	11,722,260	21	8	6.2 × 10^−7^
*At3g17185*	*TAS3*	3	miR390	5,682,143	5,682,394	80	14	3.4 × 10^−7^

n: Number of distinct alignments

k: Number of phased alignments, based on hypergeometric distribution

*: Reported *PHAS* locus

Loci with *p* < 10^−4^ are listed

Since both miRNAs and siRNAs can target mRNAs for cleavage [19, 29–31], we aimed to identify sRNA–target pairs that may be involved in photomorphogenic development. We performed degradome sequencing followed by CleaveLand analyses [32] to identify target mRNAs cleaved by expressed sRNAs in de-etiolating seedlings. The degraded mRNA samples were obtained from seedlings grown under dark (W0 h) or light (equally mixed from samples treated with W for 1, 3, 6, 12 and 24 h). Approximately 50 million reads for each of the libraries were obtained; 81–85% could be mapped to TAIR10 cDNAs (Additional file 1: Table S1). Our analyses suggested that 262 non-redundant sRNAs could mediate the cleavage of 306 *Arabidopsis* mRNAs (a total of 335 target cleavage sites). Among them, 142 cleavage events were mediated by miRNAs, 13 by phasiRNAs and 180 by other siRNAs (Fig. 1; Additional file 1: Table S5 and S6). These newly identified sRNA-target pairs are potential players in post-transcriptional gene expression regulation in *Arabidopsis* photomorphogenesis.

### sRNA abundance determines the likelihood of target mRNA cleavage

When analyzing the mRNA degradomes, we noticed that although 90 miRNA families were expressed in de-etiolating seedlings (Additional file 1: Table S2), target cleavage was detected for only members of 49 miRNA families (Additional file 1: Table S5). The results prompted us to investigate factors affecting target cleavage or the identification of degradome signatures. Previous reports have shown that high target abundance will compromise the repression activity of miRNAs and siRNAs when introduced via transfection in animal cell lines [33]. A computational model based on fixed concentration of miRNAs and mRNAs implicated that the concentration of miRNAs has a greater effect on miRNA-mRNA interaction in *Drosophila melanogaster* and in human [34]. In contrast to the seed pairing seen in most animal miRNA-mRNA interactions, most plant miRNAs interact with their target mRNAs at high complementarity that leads to cleavage of target mRNAs [19]. The availability of transcriptome data for mRNAs [12], sRNAs and degradome signatures in this study allowed us to investigate whether miRNAs/siRNAs or target abundance is important for effective miRNA/siRNA-mediated target cleavage in de-etiolating *Arabidopsis* seedlings.

**Fig. 2:**
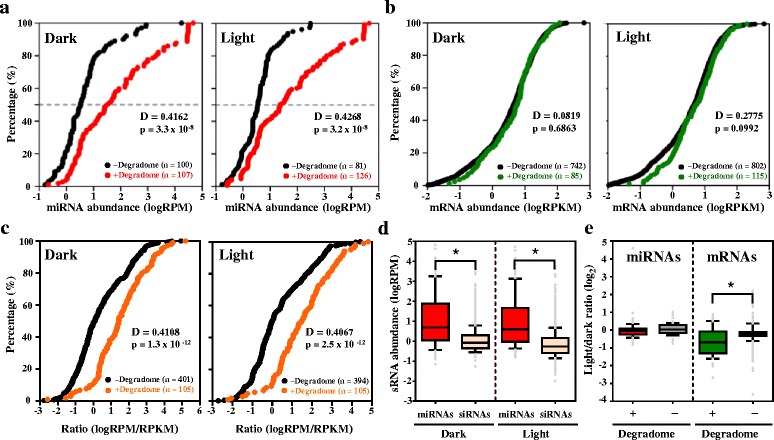
The miRNA abundance, rather than target mRNA abundance, is the major determinant of target cleavage. **a** miRNAs causing target cleavage tend to have higher abundance. Kolmogorov-Smirnov (KS) plot showing the abundance of expressed miRNAs in dark and light (W3 h). + Degradome (*red*) indicates miRNAs with valid target cleavages identified; − degradome (*black*) indicates expressed miRNAs without target cleavage identified. Dashed lines indicate 50th percentile of sRNAs. **b** Target mRNA abundance was relatively unchanged regardless of being cleaved. KS plot showing the abundance of target mRNA with (*green*) or without (*black*) degradome signatures under dark or light (W4 h). Only expressed target genes with RPKM > 0.01 were plotted. **c** Degradome signatures were preferentially detected in mRNAs with high miRNA-to-target ratios. KS plot showing distribution of pairwise miRNA-to-target ratios in dark and light (W3 h/W4 h). + Degradome (*orange*) indicates miRNA–target pairs with target cleavage identified; − degradome (*black*) indicates miRNA–target pairs without target cleavage identified. **d** The abundance is relatively lower for siRNAs than expressed miRNAs. **e** Target mRNAs, but not miRNAs, are downregulated by light. Light/dark ratios indicate the relative levels of miRNAs (W3 h/W0 h) and mRNAs (W4 h/W0 h). + and – degradome indicate miRNAs/targets with or without identified degradome signatures, respectively. Data are the mean of all biological replicates (three replicates for sRNAs and two for mRNAs). * *p* < 0.01 by Student’s t-test. The bottom, middle, and top of the box represent the 25th, 50th, and 75th percentiles, and whiskers are the 10th and 90th percentiles, respectively

Our analysis indicated that miRNAs causing target cleavage tended to have higher abundance, as compared with miRNAs that failed to generate detectable target degradome signatures (Fig. 2a). The results were similar for miRNAs expressed in both the dark (*p* = 5.2 × 10^−8^, D = 0.4327 in Kolmogorov-Smirnov test, K-S test) and light (*p* = 3.0 × 10^−8^, D = 0.4394, K-S test) (Fig. 2a). In contrast, for mRNAs with predicted miRNA target sites, the transcript abundance was comparable for mRNAs with or without degradome signatures identified (Fig. 2b). Pair-wise examination of miRNA-to-target ratios revealed relatively higher ratios for miRNA-mRNA pairs with observed cleavage under both dark (*p* = 1.3 × 10^−12^, D = 0.4108, K-S test) and light (*p* = 2.5 × 10^−12^, D = 0.4067, K-S test) (Fig. 2c). Therefore, miRNAs with high abundance may give rise to high miRNA-to-target ratios, thereby leading to successful target mRNA cleavage.

The above notion remains true when applied to siRNA-mediated mRNA cleavage (Additional file 2: Figure S1), although not as significant as for miRNAs (Fig. 2a). Among the 4255 expressed siRNAs, only 180 have degradome signatures identified for their target mRNAs (Additional file 1: Table S6), as compared with 155 targets resulting from 265 miRNA/phasiRNA-mediated cleavages (Additional file 1: Table S5), possibly because of the significantly lower expression of most siRNAs than miRNAs (Fig. 2d).

Although the expression of most of the miRNAs remained unchanged before and after light treatment, light appears to down-regulate the expression of target mRNAs with degradome signatures but not that of mRNAs without evidence of cleavage (Fig. 2e). Therefore, instead of regulating miRNA expression, light signals may potentiate the target-cleavage activities of miRNAs to tune down the expression of their target genes during de-etiolation. Whether this reduction is achieved by regulating the expression or enzymatic activities of slicer complexes remains to be investigated.

### Light optimizes miRNA functioning via the action of miR168

**Fig. 3:**
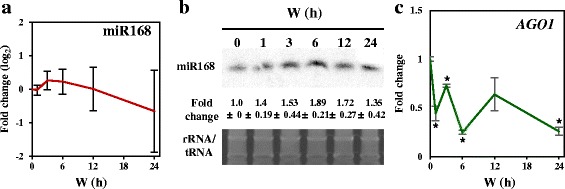
The expression of miR168 and *AGO1* in de-etiolating *Arabidopsis*. **a** Expression pattern of miR168. Data are mean ± SD from three biological replicates of sRNA sequencing. **b** Northern blot analysis of miR168 level during the times examined. Data are mean ± SD are calculated from three biological replicates. SYBR-gold stained rRNA/tRNA was a loading control. **c** Light down regulates *AGO1*. qRT-PCR results were shown as mean ± SD calculated from three technical repeats. Asterisks indicate *p* < 0.01 in Student’s t-test. Three biological replicates have shown similar results

Previously, we reported a feedback regulation between *HY5* and *HEN1*, which indicates that an sRNA equilibrium is required during photomorphogenic development [25]. Here, we sought to identify whether light regulates steps in addition to *HEN1* in sRNA biogenesis and functioning. In *Arabidopsis*, miR168 targets the sRNA slicer gene *AGO1* [35]. Deep sequencing and northern blot results indicated that the expression pattern of miR168 only slightly fluctuated under light (Fig. 3a, b). However, the mean abundance of miR168a/b ranged from 880 to 1270 read per million reads (RPM) (Additional file 1: Table S2), which is more than 10 times greater than the median level of miRNAs overall (Fig. 2a). Thus, miR168a/b has high potential in mediating the cleavage of *AGO1* transcript. Indeed, under light, *AGO1* cleavage signatures could be detected (Additional file 1: Table S5), which led to the down-regulation of *AGO1* (Fig. 3c). The detection of *AGO1* cleavage signature only under light is also consistent with preferential light-mediated downregulation of miRNA target mRNAs (Fig. 2e). Thus, miR168a/b have potential to desensitize the sRNA actions by targeting *AGO1* for degradation during photomorphogenesis.

### Light regulates the expression of some miRNAs and phasiRNAs

**Fig. 4:**
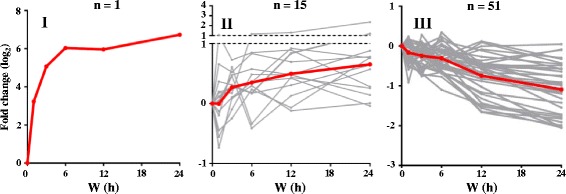
Cluster analysis of light-regulated miRNAs. Student’s t-test was performed to identify miRNAs with significant fold changes against dark (W0 h). The expression patterns of miRNAs in response to light treatments were classified into four clusters by use of k-mean clustering (by Euclidean distance). *Gray lines* indicate the average fold change of each miRNA from three biological replicates; red lines indicate average fold changes within the cluster

Although most miRNA levels were unchanged before and after light treatment in young *Arabidopsis* seedlings (Fig. 2d; Additional file 1: Table S2), we still observed that 32% (67 of 207) of expressed miRNAs were regulated by light (Fig. 4; Additional file 1: Table S2). Only 8 of 58 expressed phasiRNAs were differentially regulated by light (Additional file 1: Table S3). Because sRNA abundance is a major determinant for target cleavage in seedlings (Fig. 2), any changes in sRNA levels under light may alter their target suppression capacity. Thus, the light regulation of miRNAs and phasiRNAs may provide a timely control of target mRNAs to shape photomorphogenic development. The light responsiveness of the 67 light-regulated miRNAs could be classified into 3 major clusters by k-mean clustering (Fig. 4; Table 2). miR163 belongs to cluster I, whose expression is barely detectable in the dark but is rapidly induced by light. miR163 has been shown to promote seed germination and primary root growth during early seedling development, but not involved in light-induced inhibition of hypocotyl elongation [36]. Cluster II miRNAs are also upregulated by light, and the up regulation is more prominent after prolonged light exposure. The miRNAs in cluster II include miR157d, reported to target *HY5* during photomorphogenic development [25]. The miRNAs in cluster III were down regulated by light, especially after 6-h light exposure.

**Table 2:** Differentially expressed miRNAs in de-etiolating *Arabidopsis* seedlings, with average log_2_ fold-change listed

Cluster I	W0 h	W1 h	W3 h	W6 h	W12 h	W24 h
miR163	0	3.230582	5.070049	6.036598	5.963276	6.724631
Cluster II	W0 h	W1 h	W3 h	W6 h	W12 h	W24 h
miR5026	0	−0.19729	0.609021	−0.33921	0.435821	0.256526
miR825	0	−0.55657	−0.0974	0.484967	0.475439	0.143117
miR869.2	0	−0.44551	−0.24714	0.236649	−0.06937	0.766993
miR156c-3p	0	0.008901	0.458674	−0.41258	0.129615	0.571201
miR170-3p	0	0.199128	0.233507	0.359504	0.420726	−0.0398
miR2933a	0	−0.73393	0.087544	0.296233	−0.12048	0.00611
miR2933b	0	−0.73393	0.087544	0.296233	−0.12048	0.00611
miR5642a	0	−0.12856	0.102902	0.354152	0.920248	0.760898
miR157d	0	0.366314	0.311192	0.17222	0.304513	0.640368
miR167a-3p	0	0.046902	0.474899	0.574704	0.480747	0.881156
miR833b	0	1.053165	0.727714	0.849033	0.892631	0.279323
miR858a	0	0.657713	0.522465	0.833586	0.69362	0.780516
miR157a-3p	0	0.119139	0.300398	0.181048	0.842913	1.197978
miR157b-3p	0	0.112265	0.292249	0.169389	0.833254	1.186319
miR157c-3p	0	0.18009	0.174448	1.165619	1.299035	2.35774
Cluster III	W0 h	W1 h	W3 h	W6 h	W12 h	W24 h
miR158b	0	0.068148	−0.23119	0.046673	−0.49671	−0.77436
miR162a-3p	0	0.216902	−0.35089	−0.001	−0.53321	−1.32163
miR390a-5p	0	0.041912	−0.24039	−0.52191	−0.83531	−0.94731
miR398c-3p	0	0.233205	−0.74225	0.337007	−0.5905	−1.54736
miR399b	0	−0.32628	−0.03547	−0.57065	−0.81218	−1.16872
miR399c-3p	0	−0.13086	0.021596	−0.5015	−0.75814	−1.10903
miR829-3p.1	0	−0.19326	−0.0353	−0.14504	−0.74384	−0.90991
miR829-5p	0	0.017165	−0.29223	−0.32674	−0.66067	−0.74456
miR162b-3p	0	0.215734	−0.35239	−0.00193	−0.53434	−1.32203
miR162b-5p	0	0.057888	−0.1911	0.175723	−0.742	−1.07194
miR169a-3p	0	−0.35672	−0.10399	−0.09992	−0.43079	−0.99436
miR173-5p	0	−0.04189	−0.26578	−0.30136	−0.5118	−0.95313
miR390b-5p	0	0.041759	−0.2396	−0.52135	−0.83081	−0.94673
miR396b-3p	0	−0.19923	−0.26739	−0.52009	−0.66862	−1.11049
miR398b-3p	0	0.233205	−0.74225	0.337007	−0.5905	−1.54736
miR823	0	−0.25067	0.109541	−0.45625	−0.76484	−0.99271
miR839-5p	0	−0.9158	0.276875	−0.35478	−0.56659	−1.1664
miR166a-3p	0	0.141355	0.079493	−0.11147	−0.50323	−0.56368
miR166b-3p	0	0.158125	0.078693	−0.10249	−0.52608	−0.57401
miR166c	0	0.157754	0.078204	−0.10279	−0.52633	−0.57431
miR166d	0	0.1578	0.078219	−0.1028	−0.52637	−0.5743
miR166e-3p	0	0.157828	0.07835	−0.10235	−0.52615	−0.57403
miR166f	0	0.157829	0.078349	−0.10233	−0.5261	−0.574
miR166g	0	0.157513	0.077882	−0.10266	−0.52644	−0.57431
miR169b-3p	0	0.046702	0.084603	−0.51457	0.08493	−0.43209
miR169i	0	−0.21387	0.036407	−0.3923	−0.08524	−0.29972
miR171c-5p	0	−0.24785	−0.51146	−0.30205	−0.45579	−0.08671
miR319b	0	0.101478	0.007334	0.230599	−0.43223	−0.64435
miR393b-3p	0	−0.22189	−0.24646	−0.14695	−0.49096	−0.49076
miR408-3p	0	−0.24897	−0.16535	0.122217	−0.11517	−0.57962
miR5644	0	−0.46437	−0.21745	−0.08393	−0.26968	−0.45617
miR156h	0	−0.44634	−0.37253	−0.19148	−0.36689	−0.10394
miR160c-3p	0	−0.17658	−0.29051	−0.33976	−0.00825	0.041016
miR169m	0	−0.12627	0.064869	−0.27125	0.118007	−0.32789
miR166a-5p	0	−0.14566	−0.30587	−0.69082	−1.16879	−1.42452
miR395e	0	−0.52798	−0.63659	−0.291	−1.43631	−1.91425
miR5646	0	−0.55674	−0.18855	−0.6301	−1.65092	−1.82573
miR166b-5p	0	−0.14566	−0.30587	−0.69082	−1.16879	−1.42452
miR166e-5p	0	−0.49789	−0.68724	−1.10023	−1.47964	−1.73891
miR390b-3p	0	−0.32255	−0.76324	−0.65686	−1.66625	−1.99135
miR395a	0	−0.52798	−0.63659	−0.29058	−1.43631	−1.91425
miR395b	0	−0.30231	−0.53107	−0.43895	−1.02895	−2.04242
miR395c	0	−0.30231	−0.53107	−0.43895	−1.02895	−2.04242
miR395d	0	−0.52798	−0.63659	−0.29058	−1.43631	−1.91425
miR395f	0	−0.30231	−0.57998	−0.43895	−1.02895	−2.04242
miR396a-3p	0	−0.16376	−0.71517	−0.7776	−1.56218	−2.04976
miR408-5p	0	−0.44974	−0.17004	−0.65167	−1.20912	−1.72316
miR5012	0	−0.37776	−0.43595	−0.68763	−1.5225	−1.78667
miR5024-3p	0	−0.59768	−0.10816	−0.63027	−1.32052	−2.05354
miR5634	0	−0.19003	−0.51725	−0.68077	−0.88666	−1.19981

### miR396 promote photomorphogenesis by tuning *GRF* levels

**Fig. 5:**
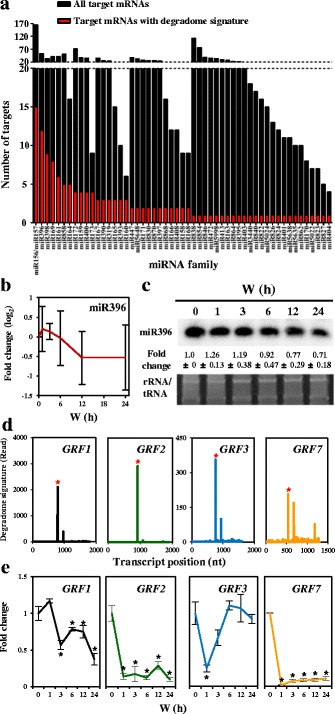
miRNA families with identified targets and the expression patterns of miR396–*GRF* regulatory pairs in de-etiolating seedlings. **a** Expressed miRNA families with predicted (*black*) or identified (*red*) target cleavages in de-etiolating *Arabidopsis*. **b** Expression of miR396 is transiently upregulated and gradually decreased on W exposure. Data are mean ± SD from three biological replicates of sRNA sequencing. **c** Northern blot analyses confirmed the expression of miR396 in de-etiolating *Arabidopsis*. One representative gel image is shown. Data are mean ± SD for the relative expression from three biological replicates. SYBR-Gold-stained rRNA/tRNA was a loading control. **d** Degradome T-plot marked the miR396-mediated *GRF1/GRF2/GRF3/GRF7* mRNA cleavage in de-etiolating seedlings. Red asterisks indicate the degradome signatures detected at expected cleavage sites for *GRF1, GRF2, GRF3* and *GRF7*. **e** Light regulation of *GRF1*, *GRF2*, *GRF3* and *GRF7*. Data are mean ± SD from three technical replicates of one representative qRT-PCR experiment. * *p* < 0.01 in Student’s t-test. Three biological replicates were performed with similar results

In de-etiolating seedlings, the degradome signatures were most frequently found for mRNAs targeted by members in the miR156/157 and miR396 families (Fig. 5a). miR157d directly targets *HY5* to desensitize the light signals during photomorphogenesis [25], but the functions of miR396 in photomorphogenic development remain obscure.

The expression of miR396 was slightly decreased upon light treatment (Fig. 5b and c). miR396 can target 7 *GROWTH REGULATING FACTOR*s (*GRF*s) [37, 38], and the cleaved signatures of all 7 *GRF*s were detected in our degradome analysis (Additional file 1: Table S5). *GRF1*, *GRF2*, *GRF3* and *GRF7* showed relatively higher degradome signature reads amongst the *GRF*s (Fig. 5d), and all showed clear down regulation under light (Fig. 5e). *GRF1*, *GRF2* and *GRF3* cooperatively regulate leaf and cotyledon development [39], whereas *GRF7* is a transcriptional repressor of abscisic acid and osmotic stress-responsive genes [40]. However, their functions in photomorphogenesis remain unknown.

To assay the regulatory roles of miR396–*GRF* pairs in photomorphogenesis, we first isolated and analyzed the *mir396a* single mutant (SALK_064047; Additional file 2: Figure S2a). Although overall miR396 levels were reduced, the phenotypes of the *mir396a* mutant were indistinguishable from that of the wild type under dark or light (Additional file 2: Fig. S2a and b). Possibly, the residual amount of miR396b in *mir396a* is sufficient for normal seedling development (Additional file 2: Figure S2a).

**Fig. 6:**
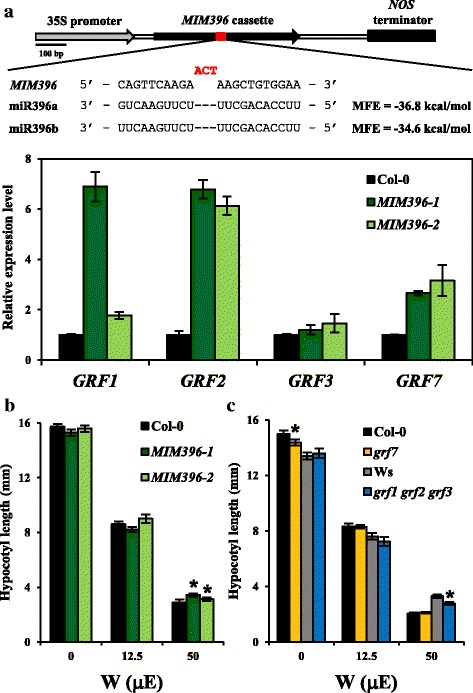
miR396 positively regulate photomorphogenesis by suppressing *GRF* levels. **a** Illustration of the *35S:MIM396* (*MIM396*) target mimicry construct. The nucleotides generating a bulge at the miR396 target site is highlighted in *red*. Minimum free energy (MFE) for *MIM396* binding to miR396a and miR396b was calculated by RNAHybrid. The expression of *GRF1*, *GRF2*, *GRF3* and *GRF7* is increased in the two independent *MIM396* T_4_ lines. Data are mean ± SD calculated from three technical replicates. Three biological replicates were performed with similar results. **b** The *MIM396* T_4_ homozygous lines show long hypocotyl length under W at 50 μE. Data are mean ± SE of hypocotyl length for one representative result. * *p* < 0.01 by Student’s t-test, *n* ≥ 30. Three biological replicates were performed with similar results. **c** The *grf1 grf2 grf3* triple mutant and the *grf7* single mutant shows shorter hypocotyl than their corresponding wild types, Ws and Col-0, under W and dark conditions, respectively. * *p* < 0.01 by Student’s t-test, *n* ≥ 30. Data are one representative result from three biological replicates performed with similar results

Because both miR396a and miR396b can suppress *GRFs* [38], we sought to simultaneously sequester these two miRNAs by generating target mimicry lines (*MIM396*) (Fig. 6a). The levels of *GRF1*, *GRF2*, *GRF3* and *GRF7* were indeed increased in two independent *MIM396* lines (Fig. 6a). The *MIM396* lines showed elongated hypocotyl length under 50 μE white light (Fig. 6b), which suggests that functional miR396 can positively regulate photomorphogenesis. We also examined the hypocotyl lengths of the *MIR396A* overexpression line (*MIR396Aox*) [38] and found that light sensitivity was not further exaggerated (Additional file 2: Figure S2c and d), so the endogenous miR396 pool may be at a saturated level for its functions in light responses.

To further understand the mechanistic roles of miR396 in photomorphogenesis, we examined the hypocotyl lengths of *grf1 grf2 grf3* and *grf7* mutants. Compared to wild-type Ws, the *grf1 grf2 grf3* triple mutant showed short hypocotyl under 50 μE white light, which indicates that the three *GRF*s act as negative regulators of photomorphogenesis. In contrast, *grf7* showed a short hypocotyl only under dark (Fig. 6c). Together with the quick repression of *GRF7* expression by light (Fig. 5e), the major function of *GRF7* is likely to promote hypocotyl elongation under dark. In sum, miR396 can act as a positive regulator of hypocotyl elongation by suppressing the negative regulator *GRF*s*.*

### Varied length and target properties of siRNAs in *Arabidopsis* seedlings

**Fig. 7:**
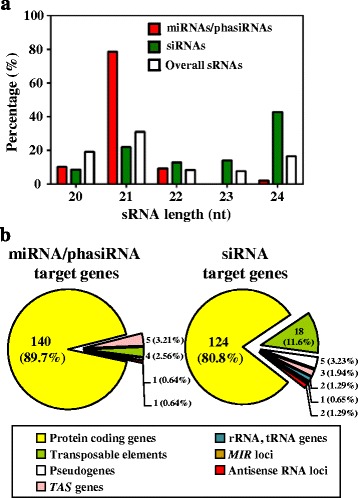
siRNA sizes and target gene features in de-etiolating seedlings. **a** Length distribution of sRNAs with targeted cleavage signatures identified (*red* and *green*). **b** Categorization of mRNAs with degradome signatures from targeted cleavage by miRNAs/phasiRNAs or siRNAs

Among the expressed siRNAs in de-etiolating seedlings, 164 are capable of mediating target cleavage (Additional file 1: Table S6). Among them, 70 (> 40%) are 24 nt long (Fig. 7a), which is quite different from the typical 21- or 22-nt miRNAs/phasiRNAs that mediate target mRNA cleavage [14, 19, 41]. Most target genes with degradome signatures for both miRNAs/phaiRNAs and siRNAs are protein coding genes (Fig. 7b). Intriguingly, 30 cleavage events from the actions of siRNAs were identified from 18 transposable elements (TEs), which is significantly higher than the number targeted by miRNAs/phasiRNAs (*p* = 3.2 × 10^−3^ by Fisher’s exact test). Most siRNA-targeted transposons are in the gypsy-like retrotransposon and CACTA-like transposase family. Eleven sRNAs that mediate TE mRNA cleavage are also derived from annotated TEs, with 4 sRNAs derived from their target loci (Additional file 1: Table S6), so these TEs may be capable of self-suppressing through TE-derived siRNAs and self-targeted cleavage. Among the 30 cleavage events derived from TE mRNAs, 23 potentially resulted from cleavage mediated by 24-nt siRNAs. The 24-nt siRNAs derived from transposable elements can mediate silencing of their original transposable elements via RNA-dependent DNA methylation (RdDM) [17, 18]. Our results suggest that in addition to RdDM, siRNA-mediated cleavage may function as an additional mechanism to prevent TE mRNA accumulation, which may escape from incomplete RdDM.

## Discussion

### Light regulates miRNA-target pairs in photomorphogenic development

In addition to the previously discovered miR157d–*HY5* and miR319–*TCP* pairs [25], we found additional miRNA–target pairs that positively regulate photomorphogenesis. In this study, miR168 could tune down *AGO1* level under light (Fig. 3), which can counteract the actions of sRNAs stabilized by the light-induced *HEN1* expression. miR396 can act as positive regulators of photomorphogenesis by suppressing *GRF1*, *GRF2*, *GRF3* and *GRF7*. The *grf7* mutant shows shorter hypocotyl length under dark, so it may positively regulate hypocotyl elongation under dark. Under light conditions, the *grf7* mutant phenotype is comparable to that of the wild type possibly because light also markedly represses *GRF7* expression (Fig. 5e, Additional file 1: Table S8).

We cannot rule out that light down-regulates the expression of *GRFs* at the transcriptional level. However, the detection of the miR396-mediated cleavage events on *GRFs* (Additional file 2: Table S5) suggested that miR396 indeed functions to optimize the *GRF* mRNA levels during de-etiolation. GRFs are known as transcription activators [42]; hence, future investigation of GRF downstream genes will help demystify genes regulated by the miR396-*GRF* lineage and provide a future research direction for their contribution in photomorphogenic development.

### sRNAs regulate photomorphogenesis from multiple angles

Our results in Fig. 2 and Additional file 2: Figure S1 showed that abundant sRNAs have a better likelihood of mediating target mRNA cleavage during photomorphogenic development. Also, despite no negative correlation between the expression of miR168/miR396 and their target mRNAs (Figs. 3 and 5), degradome signatures from their target mRNAs were observed (Additional file 1: Table S5, Fig. 5). This finding indicated that although light does not affect the accumulation of miR168 and miR396, these miRNAs can contribute to the expression repression of their target mRNAs in de-etiolating seedlings. The steady-state mRNA transcriptome during photomorphogenic growth likely is a finely orchestrated balance between the well-studied transcriptional regulation by light signals and target mRNA cleavage mediated by small regulatory RNAs as examples shown in this study.

**Fig. 8:**
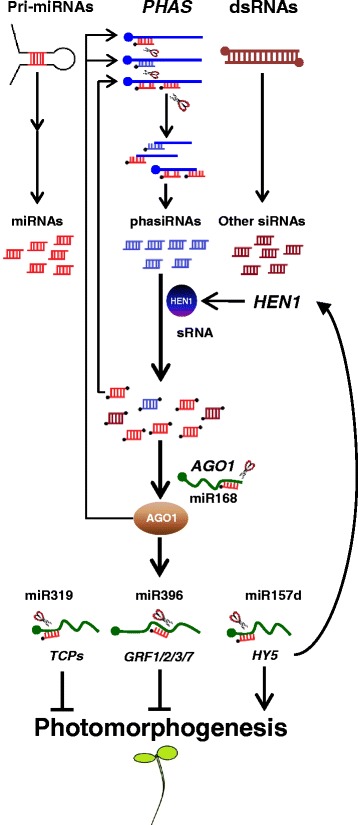
A proposed model for sRNA-mediated gene expression regulation during photomorphogenesis

Combined with our previous [25] and current discovery, sRNAs could fine-tune the expression of both positive (*HY5*) and negative (*TCPs*, *AGO1*, *GRF*s) regulators of photomorphogenesis (Fig. 8). Clearly, as key regulators of these complex and interlocked regulatory circuits, the whole plethora of sRNAs is crucial for an optimal transcriptome during photomorphogenesis. This observation also explains why mutations of single *MIR* or target gene usually show less prominent phenotypic changes (Fig. 6), as compared with mutants with a defective miRNA pathway [23–25, 30, 43–46]. We have observed a considerable amount of degradome signatures that were predicted to be results of siRNA-mediated mRNA cleavage. This suggests that, in addition to miRNAs, siRNAs also contribute considerably to down regulate their target mRNAs in de-etiolating seedlings. Further investigation of the miRNA- and siRNA-target pairs will continue to shed light on post-transcriptional regulation of photomorphogenic growth.

Finally, our observation suggests that siRNA-mediated TE mRNA cleavage may serve as an additional mode of TE silencing (Fig. 7). In *Arabidopsis*, TE mRNAs could also be cleaved by miR859 [47] and a tRNA-derived small RNA via the association with AGO1 in pollens [48]. In *Drosophila* germ cells, Piwi-interacting RNAs (piRNAs) can interact with Aubergine (Aub) or AGO3 for the cleavage of TE mRNAs [49, 50]. It remains to be clarified with which AGO protein(s) siRNAs interact for silencing plant TE mRNAs.

## Conclusions

Photomorphogenesis is a coordinated result of gene expression regulation at multiple levels. Our analyses revealed multiple sRNA–mRNA pairs contributing to this important development process. We also confirmed a comprehensive impact of sRNAs on regulating post-transcriptional gene expression during de-etiolation in *Arabidopsis*. sRNAs target multiple positive and negative regulators of photomorphogenesis, offering sophisticated fine-tuning power for regulating gene expression during de-etiolation. The potency of an sRNA in target cleavage is primarily determined by its abundance, adding an extra regulation dimension in addition to target recognition.

## Methods

### Plant materials and growth conditions

Seeds of wild-type *Arabidopsis*, Col-0, L*er*, Ws, T-DNA insertion lines SALK_064047 (*mir396a*) and SAIL_1256_F08 (*grf7*) were acquired from stock centers, ABRC or NASC. Homozygous lines of T-DNA insertion lines were screened and confirmed for phenotype observation. The *grf1 grf2 grf3* triple mutant and *35S:MIR396aox* lines were kindly provided by Drs. Jeong Hoe Kim and DiQiu Yu, respectively. For phenotype observation, *Arabidopsis* seeds were surface-sterilized with 30% bleach and sown on half-strength Murashige and Skoog medium (Duchefa) without supplementing vitamin or sucrose, with 0.8% phyto agar at tissue culture grade (Duchefa, CAS number 9002–18-0). Seeds were stratified (4 °C for 4 days in the dark) to synchronize germination, then exposed to white light for 1 h to stimulate germination, and transferred to different light (W) conditions at 22 °C (Dark, W 12.5 and 50 μE for 4 days). The white light source was a PHILIPS LIFEMAX T-LD 18 W/840 T25 cool white tube. Hypocotyl lengths of seedlings were measured by using ImageJ v1.47 [51]. The means and SEM were calculated from more than 30 seedlings. At least 3 biological replicates for each line were used for each experiment.

### Construction of *MIM396* lines

Primer sequences used in this study are listed in Additional file 1: Table S10. *35S:MIM396* target mimicry lines were generated as described [52]. Briefly, the genomic fragment of *IPS1* was amplified by using the iProof High-fidelity PCR kit (Bio-Rad) and cloned into the pGEMT-easy vector (Promega). The miR399 target site on original *IPS1* sequence was modified to sequester miR396a/b, as shown in Fig. 6a, by overlapping PCR during construction [52]. All constructs were then subcloned into the pCambia-1390 binary vector (CSIRO) digested with *Sal*I and *Sac*I. The constructs were transformed into *Agrobacterium tumefaciens* GV3101 strain, and introduced into *Arabidopsis* Col-0 by floral dipping. Two independent homozygous transgenic lines per construct were used for further analyses.

### RNA sequencing and data analyses

For sRNA sequencing and data analyses, 4-d-old dark-grown *Arabidopsis* Col-0 seedlings were exposed to white light (100 μE) for 1, 3, 6, 12 and 24 h. The aerial tissues of approximately 5000 seedlings were collected for RNA isolation. Ten to 15 μg total RNA was size fractionated on 15% Tris-Borate-EDTA-Urea gel. sRNAs ranging from 17 to 30 nucleotides were gel-purified and used for cDNA library construction (Illumina Truseq for replicates 1 and 2, Small RNA v1.5 for replicates 3) and sequencing with the use of an Illumina HiSeq 2500. Twelve barcoded samples were sequenced in one single flowcell (a total of 240 M reads output per flowcell) at a read length of 50 nt. The adaptor-trimmed reads with size >18 nt were mapped to the *Arabidopsis* TAIR10 genome by using Bowtie [53] with the parameters -f –n 0 –e 80 –l 18 –a –m 5 –best –strata. For miRNA profiling, reads that perfectly matched to mature miRNA sequences were counted, normalized to total mapped reads of 20–24 nt and were shown as reads per million reads (RPM). Reads that mapped to miRNA families (e.g., miR156) were weighted by dividing the read count and equally assigning to each miRNA family member. For siRNA quantification, the Bowtie parameters were -f –n 0 –e 80 –l 18 –a –v 2 –best –strata. For phasiRNAs, the prediction of *PHAS* loci involved use of the UEA sRNA Workbench [54]. In brief, adaptor-trimmed reads longer than 16 nt were mapped to the TAIR10 genome, and the 21-nt phasing register was set to detect phasing within a 251-nt window, based on hypergeometric distributions described previously [14]. Among the uniquely mapped miRNA/siRNAs, only those with read counts ≥5 in ≥1 time point for all 3 biological replicates were considered expressed. Light-regulated sRNAs were defined as sRNAs with *p* < 0.05 on Student’s t-test compared to dark treatment (W0 h) for all 3 biological replicates. For mRNA transcriptome analysis, the RPKM for sRNA target genes were analyzed by using datasets published previously [12]. Potential targets included those predicted by use of psRNATarget [55] (with UPE = 25, expectation = 3), miRNA targets identified in previous studies [25, 37] and miRNA targets detected in our degradome analysis (see Additional file 1: Table S5). Expressed genes had RPKM > 0.01 in at least one time point in both biological replicates. The transcript levels of target genes with degradome signatures are in Additional file 1: Table S5 and S6.

For degradome sequencing, 100 μg total RNA was isolated from 4-d-old dark-grown seedlings and mixtures of 4-d-old dark-grown seedlings exposed to 1, 3, 6, 12 and 24 h of light. Degradome sequencing was performed as described [56–58]. Putative cleavage sites were identified by using Cleaveland v4.4.3 [32, 59]. Those with CleaveLand category ≤ 2, *p* ≤ 0.05 and at least 5 reads at the predicted cleavage site were reported as valid targeting events.

### Northern blot analysis and qRT-PCR

In total, 20 to 50 μg total RNA was separated on 15% TBE-Urea gel (Invitrogen). SYBR-Gold (Life Technologies) was used for visualizing RNAs on gels. RNAs were then transferred to Hybond-N+ Nylon membrane (GE Healthcare), by using Transblot SD Semi-Dry Transfer Cell (Bio-Rad) and hybridized with γ-^32^P-labeled miRNA probes as indicated at 37° Celsius overnight in UltraHyb Oligo buffer (Ambion). Hybridized blots were washed and exposed to Phosphoimager (GE Healthcare), then analyzed by using Typhoon FLA 7000 (GE Healthcare Life Sciences), as described [14]. Images were quantified by using ImageJ v1.47 [51]. For qRT-PCR, cDNA was synthesized from 2 μg total RNA from 4-d-old de-etiolating *Arabidopsis* seedlings. The SuperScript II RT kit (Invitrogen) was used for reverse transcription of mRNA. For qRT-PCR, cDNA with 0.25 ng equivalence of mRNA was used as a template for each sample. PCR amplification and detection was as described [60]. Primers are in Additional file 1: Table S8. Data for one representative biological replicate were shown in Figs. 3c, 5e and 6a. Results for 2 additional biological replicates were shown in Additional file 2: Figure S3.

## Additional files

Additional file 1: **Table S1.** Sequencing and mapping statistics. Description: Contains the number and percentage of mapped sRNA reads in sRNA and degradome sequencing. **Table S2.** Expressed miRNAs in de-etiolating *Arabidopsis* seedlings. Description: Contains the expression level of expressed miRNAs, with RPM and fold changes. **Table S3.** Expressed phasiRNAs in de-etiolating seedlings. Description: Contains the expression level of expressed phasiRNAs, with the corresponding *PHAS* locus, RPM and fold changes. **Table S4.** Expressed siRNAs in de-etiolating seedlings. Description: Contains the expression level of expressed siRNAs, with the sequence, length, RPM and fold changes. **Table S5.** miRNA-mRNA and phasiRNA-mRNA pairs from cross comparisons of sRNA transcriptome and mRNA degradome. Contains the information of miRNA/phasiRNA-mediated target cleavage, including locus number, CleaveLand categories, *p*-values and target mRNA levels. **Table S6.** siRNA-mRNA pairs from cross comparisons of sRNA transcriptome and mRNA degradome. Contains the information of siRNA-mediated target cleavage, including locus number, CleaveLand categories, *p*-values and target mRNA levels. **Table S7.** Expression levels of *GRF*s. Contains the expression level of *GRF*s under dark and light. **Table S8.** Primers used in this study. Contains sequences of the primers that are used in this study. (XLSX 2667 kb)

Additional file 2: **Figure S1.** miRNAs/siRNAs with target cleavage tend to be more abundant than miRNAs/siRNAs that did not show target cleavage signatures. Contains supplemental figure and legend showing K-S test results of siRNA abundance. **Figure S2.** Molecular and phenotypic analyses of *mir396a* mutant and *MIR396aox* lines. Contains supplemental figure and legend showing the examination of *mir396a* mutant and *MIR396aox* lines. **Figure S3.** qRT-PCR results of two additional biological replicates for a *AGO1* and b, c *GRF* shown in Figs. 3c, 5e and 6a, respectively. Contains supplemental figure and legend showing the qRT-PCR results of two additional biological replicates in this study. (PDF 1074 kb)

## Abbreviations

AGO1: ARGONAUTE1
Aub: Aubergine
B: blue
COP1: CONSTITUTIVE PHOTOMORPHOGENESIS 1
cry: cryptochrome
FR: far-red
GRF: GROWTH REGULATING FACTOR
HEN1: HUA ENHANCER1
HST: HASTY
HY5: ELONGATED-HYPOCOTYL 5
HYL1: HYPONASTIC LEAVES 1
K-S test: Kolmogorov-Smirnov test
miRNA or miR: microRNA
*PHAS*: phasiRNA-generating loci
phasiRNAs: phased siRNA
phy: phytochrome
piRNA: Piwi-interacting RNA
R: red
RdDM: RNA-dependent DNA methylation
RPM: read per million reads
siRNA: small interfering RNA
sRNA: small regulatory RNA
*TAS*: trans-acting siRNA-generating loci
TCP: TEOSINTE BRANCHED 1 CYCLOIDEA AND PCF TRANSCRIPTION FACTOR
TE: transposable element
