# Supplementary material for: Unraveling multifaceted contributions of small regulatory RNAs to photomorphogenic development in Arabidopsis
Source: BMC Genomics. 2017 Jul 24;18:559. doi: 10.1186/s12864-017-3937-6 (PMC5525271; doi:10.1186/s12864-017-3937-6)
Supplement: Supplementary file 2 — Figure S1. miRNAs/siRNAs with target cleavage tend to be more abundant than miRNAs/siRNAs that did not show target cleavage signatures. Contains supplemental figure and legend showing K-S test results of siRNA abundance. Figure S2. Molecular and phenotypic analyses of mir396a mutant and MIR396aox lines. Contains supplemental figure and legend showing the examination of mir396a mutant and MIR396aox lines. Figure S3. qRT-PCR results of two additional biological replicates for a AGO1 and b, c GRF shown in Figs. 3c, 5e and 6a, respectively. Contains supplemental figure and legend showing the qRT-PCR results of two additional biological replicates in this study. (PDF 1074 kb) [file 12864_2017_3937_MOESM2_ESM.pdf]

Figure S1

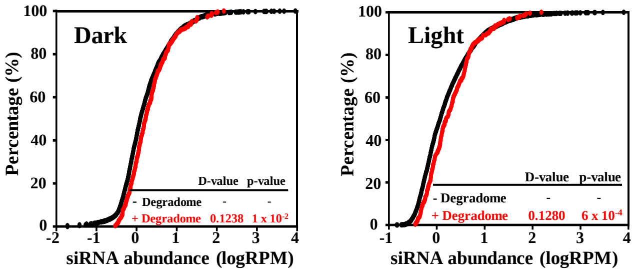

Figure S1

miRNAs/siRNAs with target cleavage tend to be more abundant than miRNAs/siRNAs that did not show target cleavage signatures. KS plot showing distribution of siRNAs. + Degradome (red) indicates sRNA with target mRNA signature identified; - degradome indicates expressed siRNAs without target signature identified in de-etiolating seedlings. Data are calculated from average of three biological replicates. p-values and D-values were calculated from KS test.

Figure S2

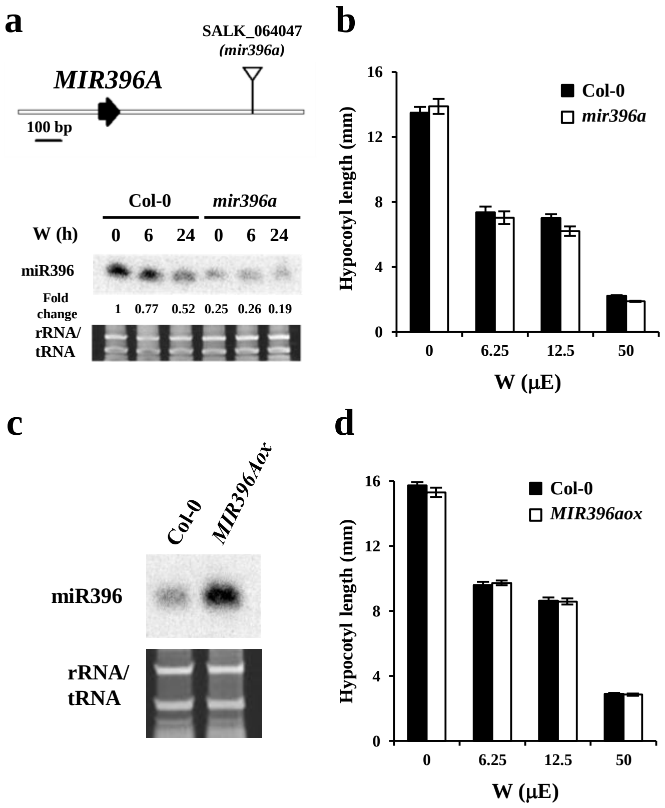

Figure S2

Molecular and phenotypic analyses of *mir396a* mutant and *MIR396aox* lines. **a** Illustration of T-DNA insertion site and the confirmation of reduced miR396 levels in *mir396a* (SALK_064047). **b** Comparable hypocotyl lengths between *mir396a* mutant and wild-type Arabidopsis under both dark and W conditions. One representative result is shown, n ≥ 30. Three biological replicates were performed with similar results. **c** Northern blot analysis of confirmed overexpression of miR396 in *MIR396aox* line. Four-d-old dark-grown seedlings were used for RNA isolation. SYBR-Gold stained rRNA/tRNA was a loading control. **d** Comparable hypocotyl lengths between *MIR396aox* line and wild-type Arabidopsis under dark and W conditions. One representative result is shown, n ≥ 30. Three biological replicates were performed with similar results.

Figure S3

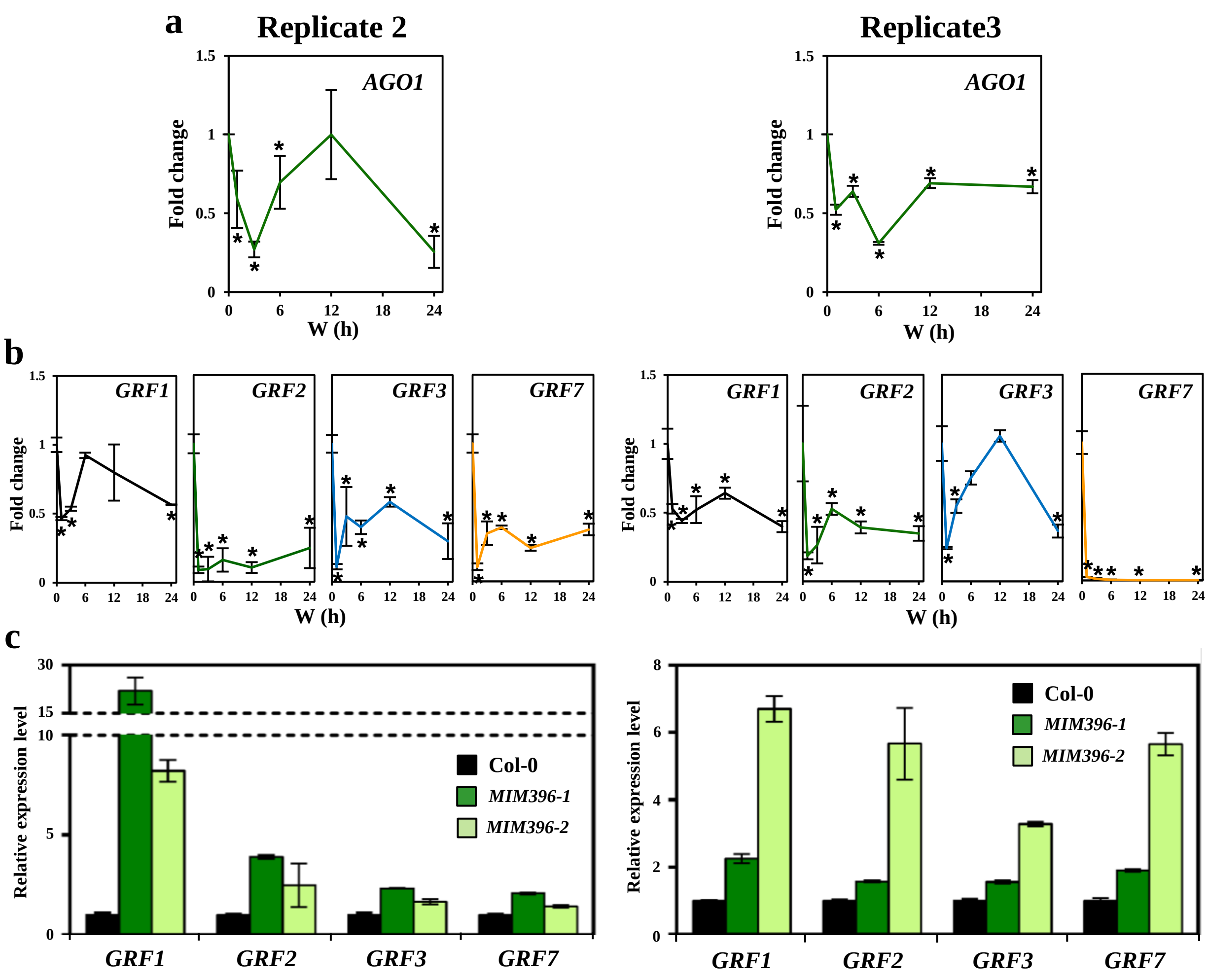

Figure S3
qRT-PCR results of two additional biological replicates for **a** *AGO1* and **b, c** *GRF* shown in Fig. 3c, 5e and 6a, respectively. Data were shown as mean ± SD from three technical replicates. Asterisks indicate $p < 0.01$ in Student's t-test.
